# Transducin β-like 1 X-linked receptor 1 suppresses cisplatin sensitivity in Nasopharyngeal Carcinoma via activation of NF-κB pathway

**DOI:** 10.1186/1476-4598-13-195

**Published:** 2014-08-22

**Authors:** Shu-Peng Chen, Qi Yang, Chan-Juan Wang, Long-Juan Zhang, Yi Fang, Fang-Yong Lei, Shu Wu, Li-Bing Song, Xiang Guo, Ling Guo

**Affiliations:** Department of Experimental Research, State Key Laboratory of Oncology in South China Guangzhou, Guangdong, 510060 People’s Republic of China; Department of Nasopharyngeal Carcinoma, State Key Laboratory of Oncology in South China Guangzhou, Guangdong, 510060 People’s Republic of China; Laboratory of General Surgery, The First Affiliated Hospital of Sun Yat-sen University Guangzhou, Guangdong, 510080 People’s Republic of China; Department of Pulmonary Function Laboratory, Intensive Care Unit, State Key Laboratory of Oncology in South China Guangzhou, Guangdong, 510060 People’s Republic of China

**Keywords:** TBL1XR1, Nasopharyngeal carcinoma, Cisplatin, Chemotherapy, Anti-Apoptotic, NF-κB signalling

## Abstract

**Background:**

Transducin β-like 1 X-linked receptor 1 (TBL1XR1) is an important transcriptional cofactor involved in the regulation of many signaling pathways, and is associated with carcinogenesis and tumor progression. However, the precise role of TBL1XR1 in these processes is not well understood.

**Methods:**

We detected the expression of TBL1XR1 protein and mRNA in nasopharyngeal carcinoma (NPC) cell lines and biopsies by western blotting, real-time PCR and immunohistochemical staining (IHC). Overexpression of TBL1XR1 in NPC enhanced chemoresistance to cisplatin using two NPC cell lines in vitro and in vivo.

**Results:**

TBL1XR1 was upregulated in NPC cell lines and clinical samples. The expression of TBL1XR1 was correlated with several clinicopathological factors including clinical stage, T classification, N classification and patient survival. Univariate and multivariate analysis revealed that TBL1XR1 was an independent prognostic factor for patient survival. *In vitro* and *in vivo* studies demonstrated that TBL1XR1 high expression induced resistance to cisplatin-induced apoptosis in NPC cells. Furthermore, we found that TBL1XR1 activated the NF-κB pathway and promoted transcription of genes downstream of NF-κB, especially anti-apoptotic genes.

**Conclusions:**

Upregulation of TBL1XR1 induces NPC cells resistance to cisplatin by activating the NF-κB pathway, and correlates with poor overall survival of NPC patients. TBL1XR1 has a pivotal role in NPC and could be a valuable prognostic factor as well as a novel biomarker for tailoring appropriate therapeutic regimes.

**Electronic supplementary material:**

The online version of this article (doi:10.1186/1476-4598-13-195) contains supplementary material, which is available to authorized users.

## Background

Nasopharyngeal carcinoma (NPC) is a common malignant carcinoma of the head and neck region, and is more prevalent in regions of Southeast Asia and Africa than elsewhere [1]. The etiological factors of NPC mainly consist of genetic susceptibility, Epstein–Barr virus (EBV) infection and environmental factors [1]. Currently, the standard treatment for NPC consists of radiotherapy and adjuvant cisplatin chemotherapy. Although this therapeutic regimen results in a high cure rate, a considerable number of patients suffer from therapeutic resistance, distant metastases and local recurrence after treatment [2–6]. Thus, it is important to understand the molecular events involved in the development and progression of NPC, with an aim to explore effective strategies that can enhance the sensitivity of tumor cells to drug-induced apoptosis.

Transducin β-like 1 X-linked receptor 1 (TBL1XR1) or transducin β-like-related protein 1 (TBLR1) was originally identified as a component of the nuclear receptor corepressor (N-CoR) complex [7]. TBL1XR1 is high homology to Transducin β-like protein 1 (TBL1); both contain F-box/WD-40 repeats that are required for binding to the silencing mediator for retinoid and thyroid hormone receptors (SMRT) and the N-CoR corepressor complex, which mediates transcriptional repression by unliganded nuclear receptors [8, 9]. TBL1XR1 also function as an E3 ubiquitin ligase that recruits UbcH5 ubiquitin conjugating enzymes/19S proteasome, and subsequently replaces of corepressors with coactivators in a ligand-dependent manner [10]. Previous studies have established TBL1XR1 as a key player in the regulation of multiple signaling pathways (Wnt/β-catenin, Notch, NF-κB, and nuclear receptor) and gene transcription [10–13]. In addition, TBL1XR1 has been found to affect carcinogenesis and tumor progression. Liu et al. showed that TBL1XR1 is overexpressed in lung cancer cells, and particularly in a human immortalized bronchial epithelial cell line, indicating that the abnormal expression of TBL1XR1 might be an early event during lung cancer development [14]. Kadota and colleagues observed that TBL1XR1 levels are amplified in breast cancer, and the protein plays an oncogenic role in breast cancer progression [15]. Furthermore, the hepatic transcriptional cofactor, TBL1/TBLR1, was found to regulate liver lipid metabolism via the nuclear receptor peroxisome proliferator-activated receptor (PPAR)α, and the deficiency of TBL1/TBLR1 activity induced liver steatosis and hypertriglyceridemia [16].

In this study, we showed that TBL1XR1 is upregulated in NPC cell lines and clinical samples, and TBL1XR1 expression levels were correlated with the clinicopathologic characteristics of NPC patients. Furthermore, TBL1XR1 induced anti-apoptotic abilities in NPC cells by activating NF-κB signaling pathway. Our data indicated that TBL1XR1 is a novel prognostic factor and may serves as an effective biomarker for selective therapeutic regimen for NPC patients.

## Results

### Up-regulation of TBL1XR1 in NPC cells

**Figure 1:**
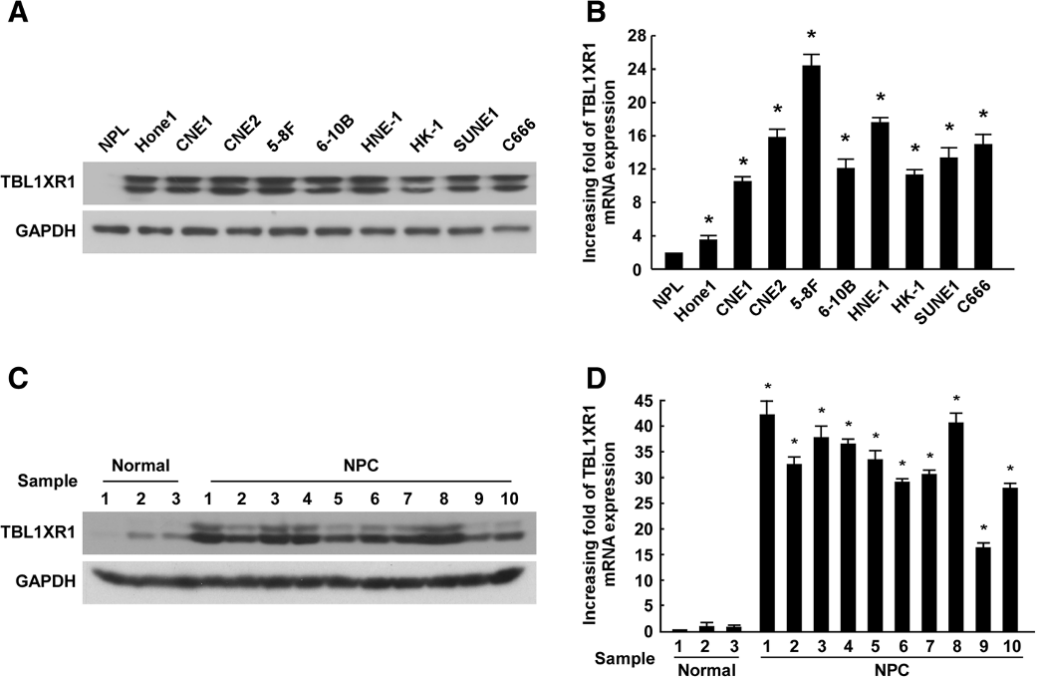
**Up-regulation of TBL1XR1 in NPC cells. (A)** Western blotting analysis of TBL1XR1 protein level in normal nasopharyngeal epithelial cells (NPEC) and 9 cultured NPC cell lines. GAPDH was used as a loading control. **(B)** Reverse transcription (RT)-PCR and real-time PCR analysis of TBL1XR1 mRNA level in normal nasopharyngeal epithelial cells (NPEC) and 9 cultured NPC cell lines. GAPDH was used as a loading control. * P ≤ 0.05. **(C)** Western blotting analysis of TBL1XR1 protein level in three normal nasopharyngeal epithelial biopsies and 10 NPC samples. GAPDH was used as a loading control. **(D)** Reverse transcription (RT)-PCR and real-time PCR analysis of TBL1XR1 mRNA level in three normal nasopharyngeal epithelial biopsies and 10 NPC samples. GAPDH was used as a loading control. * P ≤ 0.05.

Western blot analysis showed that TBL1XR1 was highly expressed in all nine NPC cell lines, whereas it was weakly detected in normal nasopharyngeal epithelial cells (NPECs; Figure 1A). Reverse transcription (RT)-PCR and real-time PCR were performed to detect and measure expression levels of TBL1XR1 mRNA. All nine NPC cell lines showed significantly higher levels of TBL1XR1 mRNA compared to NPECs (Figure 1B). This was consistent with the high levels of TBL1XR1 protein measured in NPC cells.

To validate whether the upregulation of TBL1XR1 in NPC cell lines was clinically relevant, we also examined protein and mRNA levels in NPC tissues.TBL1XR1 was found to be overexpressed to varying degrees in all 10 NPC samples (Figure 1C), and was barely detectable in the three healthy nasopharyngeal epithelial tissue samples. RT-PCR and real-time PCR revealed that TBL1XR1 mRNA was upregulated in tumor samples (Figure 1D), confirming that TBL1XR1 is overexpressed in NPC patients.

### TBL1XR1 expression correlates with clinicopathologic characteristics of NPC patients

**Figure 2:**
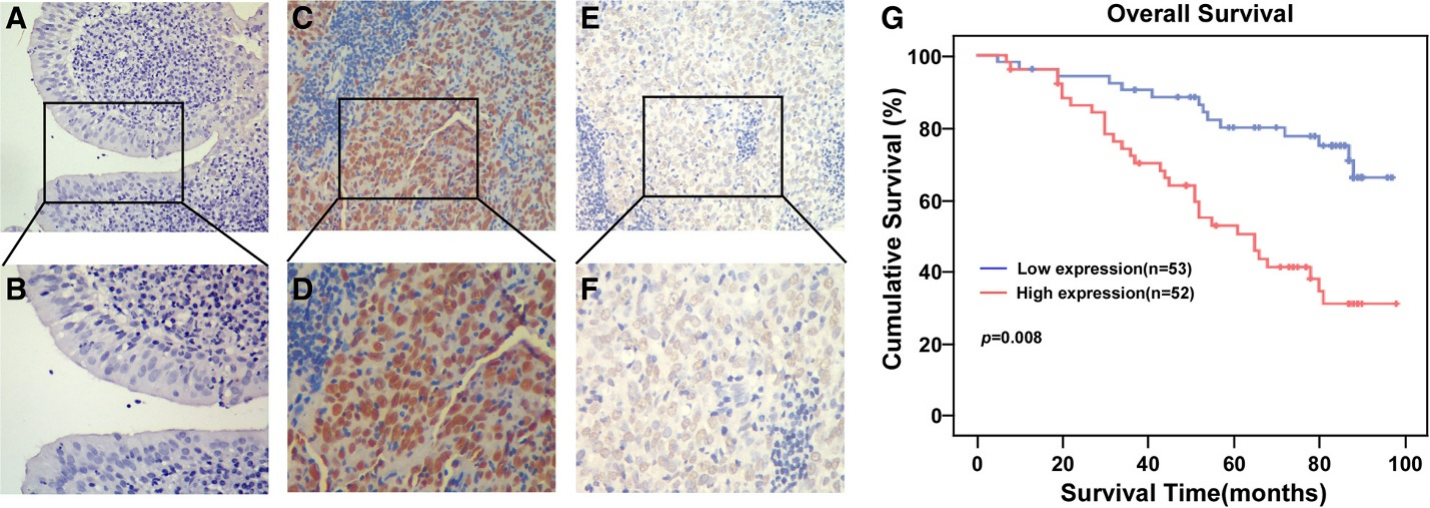
**TBL1XR1 expression correlates with clinicopathologic characteristics of NPC patients.** **(A and B)** No expression of TBL1XR1 protein in normal nasopharyngeal epithelial biopsies. **(C and D)** High TBL1XR1 expression in NPC tissues. **(E and F)** Low TBL1XR1 expression in NPC tissues. **A**, **C**, **E** (SP, ×200) and **B**, **D**, **F** (SP, ×400). **(G)** Kaplan-Meier curves with univariate analyses for patients with low TBL1XR1 expression versus high TBL1XR1 expression tumors. P values were calculated by log-rank test.

To further demonstrate TBL1XR1 protein is overexpressed in clinical samples of NPC, we performed immunohistochemical (IHC) staining on paraffin-embedded archived biopsies (105 NPC samples and 3 normal nasopharyngeal epithelial tissue samples). In agreement with the results above, TBL1XR1 was barely detected in normal nasopharyngeal epithelial tissues, while strong expression was observed in the tumor cells of NPC samples (Figure 2A). TBL1XR1 was detectable in 94 of 105 (89.52%), and high levels of expression were observed in 52 (49.52%) of the samples (Table 1).

**Table 1:** **Correlations between TBL1XR1 expression and clinicopathologic characteristics of nasopharyngeal carcinoma patients**

Characteristics	No. cases	TBL1XR1 expression		Chi-square
		Low or none	High	Test P-value
		No. cases(%)	No. cases(%)	
Gender				
Male	79	41(51.9)	38(48.1)	0.611
Female	26	12(46.2)	14(53.8)	
Age(years)				
≤45	55	27(49.1)	28(50.9)	0.766
>45	50	26(52.0)	24(48.0)	
Clinical Stage				
I	2	2(100.0)	0(0.0)	
II	21	18(85.7)	3(14.3)	0.001
III	49	22(44.9)	27(55.1)	
IV	33	11(33.3)	22(66.7)	
T classification				
T1	3	3(100.0)	0(0.0)	
T2	32	21(65.6)	11(34.4)	0.040
T3	44	19(43.2)	25(56.8)	
T4	26	10(38.5)	16(61.5)	
N classification				
N0	26	18(69.2)	8(30.8)	
N1	44	26(59.1)	18(40.9)	0.003
N2	22	5(22.7)	17(77.3)	
N3	13	4(30.8)	9(69.2)	
Patient survival				
Alive	59	39(66.1)	20(33.9)	0.001
Deceased	46	14(30.4)	32(69.6)	

We also investigated the possible correlations between TBL1XR1 expression levels and the clinicopathologic characteristics of NPC. Analysis of 105 NPC samples indicated that TBL1XR1 expression was correlated with clinical staging (P = 0.001), T classification (P = 0.040), N classification (P = 0.003), and patient survival (P = 0.001). These results show that the observed correlation between TBL1XR1 expression and NPC progression is clinically relevant.

### Five-year survival rate in NPC patients

The results of the Kaplan–Meier survival analysis and log-rank tests demonstrated that high expression of TBL1XR1 was correlated with poor prognosis in NPC patients (P = 0.008 vs. low TBL1XR1 expression, Figure 2B). The five-year overall survival rate of the group that expressed low levels of TBL1XR1 was 78.939% (95% CI: 88.935%-62.153%) compared to 59.183% (95% CI: 71.667%-44.758%) for the group expressing high levels. Multivariate Cox regression analysis indicated that TBL1XR1 was an independent prognostic factor for overall survival in NPC (Table 2), and may therefore act as a prognostic biomarker.

**Table 2:** **Univariate and multivariate analysis of different prognostic parameters in patients with nasopharyngeal carcinoma by Cox-regression analysis**

Characteristics	Univariate analysis			Multivariate analysis		
	No. cases	P	Regression coefficient(SE)	P	Relative risk	95% CI
T classification		0.001	0.198	0.010	1.753	1.146-2.683
T1	3					
T2	32					
T3	44					
T4	26					
N classification		<0.001	0.151	0.005	1.540	1.143-2.076
N0	26					
N1	44					
N2	22					
N3	13					
TBL1XR1 expression		<0.001	0.325	0.043	2.013	1.024-3.958
Low or none	53					
High	52					

### Increased TBL1XR1 expression suppresses cisplatin-induced apoptosis

**Figure 3:**
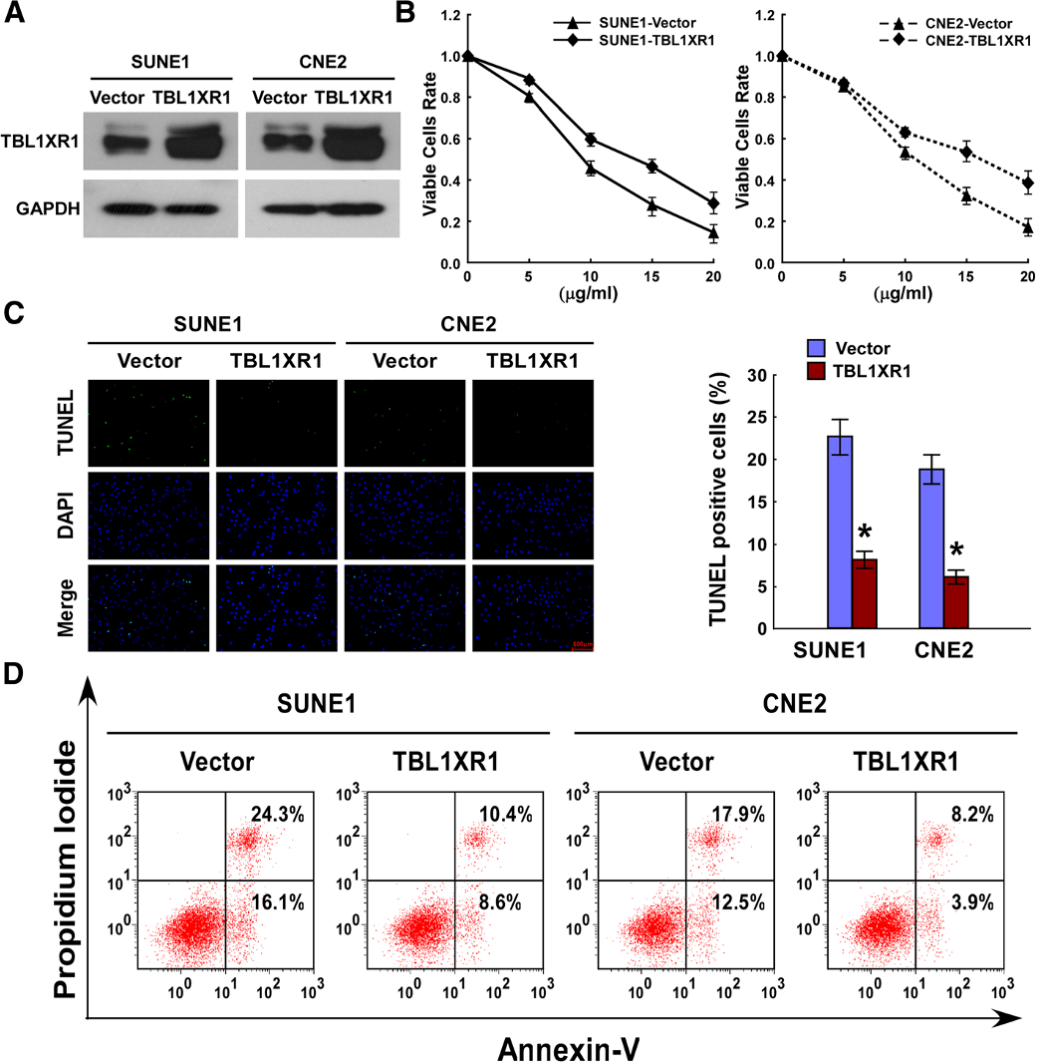
**NPC Cells overexpressing TBL1XR1 proteins are less sensitive to cisplatin-induced apoptosis. (A)** Overexpression of TBL1XR1 in NPC cell lines. Western blotting analysis of TBL1XR in SUNE1-vector, SNUE-TBL1XR1, CNE2-vector and CNE2-TBL1XR1 cells. GAPDH was used as loading control. **(B)** SUNE1-vector, SNUE-TBL1XR1, CNE2-vector and CNE2-TBL1XR1 cells treated by cisplatin (5 μg/ml, 10 μg/ml, 15 μg/ml, 20 μg/ml) for 48 hours. MTT analysis of the proportion of cells still alive after treatment. **(C)** Quantification of TUNEL positive cells. SUNE1-vector, SNUE-TBL1XR1, CNE2-vector and CNE2-TBL1XR1 cells were treated by cisplatin (20 μg/ml) for 24 h, followed by TUNEL staining and the number of TUNEL-positive cells was counted from 10 random fields of at least 500 cells. Results are expressed as percentages of total cells. **(D)** Flow Cytometry analysis of Annexin V^+^/PI^¯^ cells after the indicated cell lines treated with cisplatin (20 μg/ml) for 24 h. Results are expressed as percentages of total cells. * P ≤ 0.05.

To investigate the biological effect of TBL1XR1 in NPC progression, two NPC cell lines (SUNE1 and CNE2) were established that stably overexpressed TBL1XR1 (Figure 3A). Cells were treated with increased doses of cisplatin, a commonly used chemotherapeutic agent. The proportion of cells still alive after treatment was plotted on a survival curve. Following cisplatin treatment, control cells exhibit a marked decline in survival rate, while the decline in survival of cells that overexpressed TBL1XR1 was much reduced (Figure 3B). The results of TUNEL and Annexin-V binding assays suggested that both TBL1XR1-overexpressing cell lines exhibited enhanced resistance to cisplatin treatment (Figure 3C and D). Taken together, these data indicated that ectopic overexpression of TBL1XR1 could reduce the effectiveness of cisplatin against NPC cells.

### TBL1XR1 knockdown increases sensitivity to cisplatin-induced apoptosis

**Figure 4:**
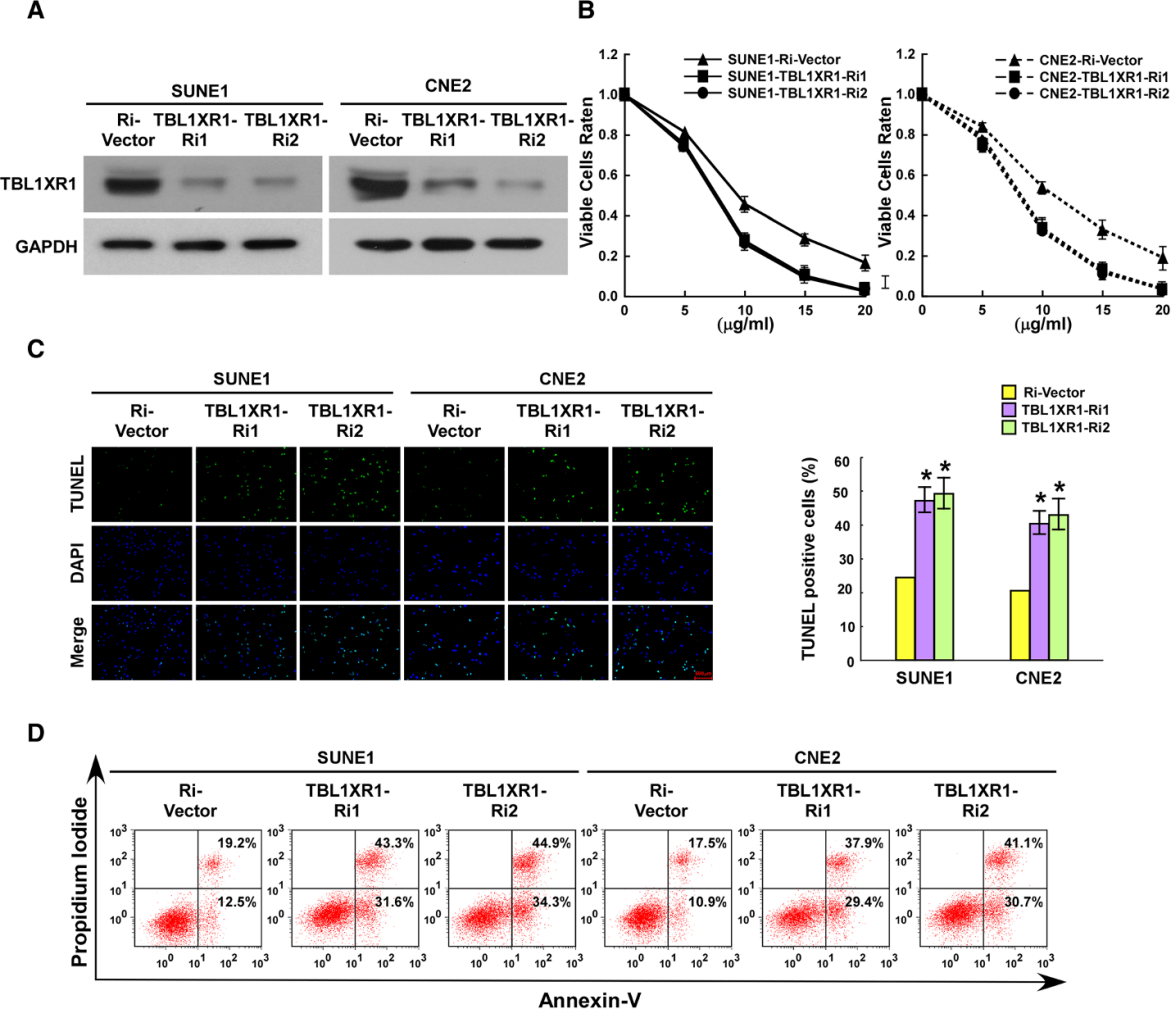
**Downregulation of endogenous TBL1XR1 expression decreases the resistance of NPC cells to apoptosis. (A)** TBL1XR1 knockdown was achieved by introducing specific shRNA in NPC cells. Western blotting analysis of TBL1XR in SUNE1-vector, SNUE-TBL1XR1-Ri, CNE2-vector and CNE2-TBL1XR1-Ri cells. GAPDH was used as loading control. **(B)** SUNE1-vector, SNUE-TBL1XR1-Ri, CNE2-vector and CNE2-TBL1XR1-Ri cells treated by cisplatin (5 μg/ml, 10 μg/ml, 15 μg/ml, 20 μg/ml) for 48 hours. MTT analysis of the proportion of cells still alive after treatment. **(C)** Quantification of TUNEL positive cells. SUNE1-vector, SNUE-TBL1XR1-Ri, CNE2-vector and CNE2-TBL1XR1-Ri cells were treated by cisplatin (20 μg/ml) for 24 h, followed by TUNEL staining and the number of TUNEL-positive cells was counted from 10 random fields of at least 500 cells. Results are expressed as percentages of total cells. **(D)** Flow Cytometry analysis of Annexin V^+^/PI^¯^ cells after the indicated cell lines treated with cisplatin (20 μg/ml) for 24 h. Results are expressed as percentages of total cells. * P ≤ 0.05.

In order to investigate the role of TBL1XR1 in apoptotic pathways, TBL1XR1 expression was silenced using specific shRNAs (Figure 4A). As expected, TBL1XR1 downregulated cells were more sensitive to cisplatin than control cells; survival curves were steeper (Figure 4B), and the quantity of viable and non-viable apoptotic cells increased (Figure 4C and D, respectively). In summary, TBL1XR1 played an important role in resistance to cisplatin treatment in NPC cells.

### TBL1XR1 suppressed the sensitivity of NPC cells to cisplatin in vivo

**Figure 5:**
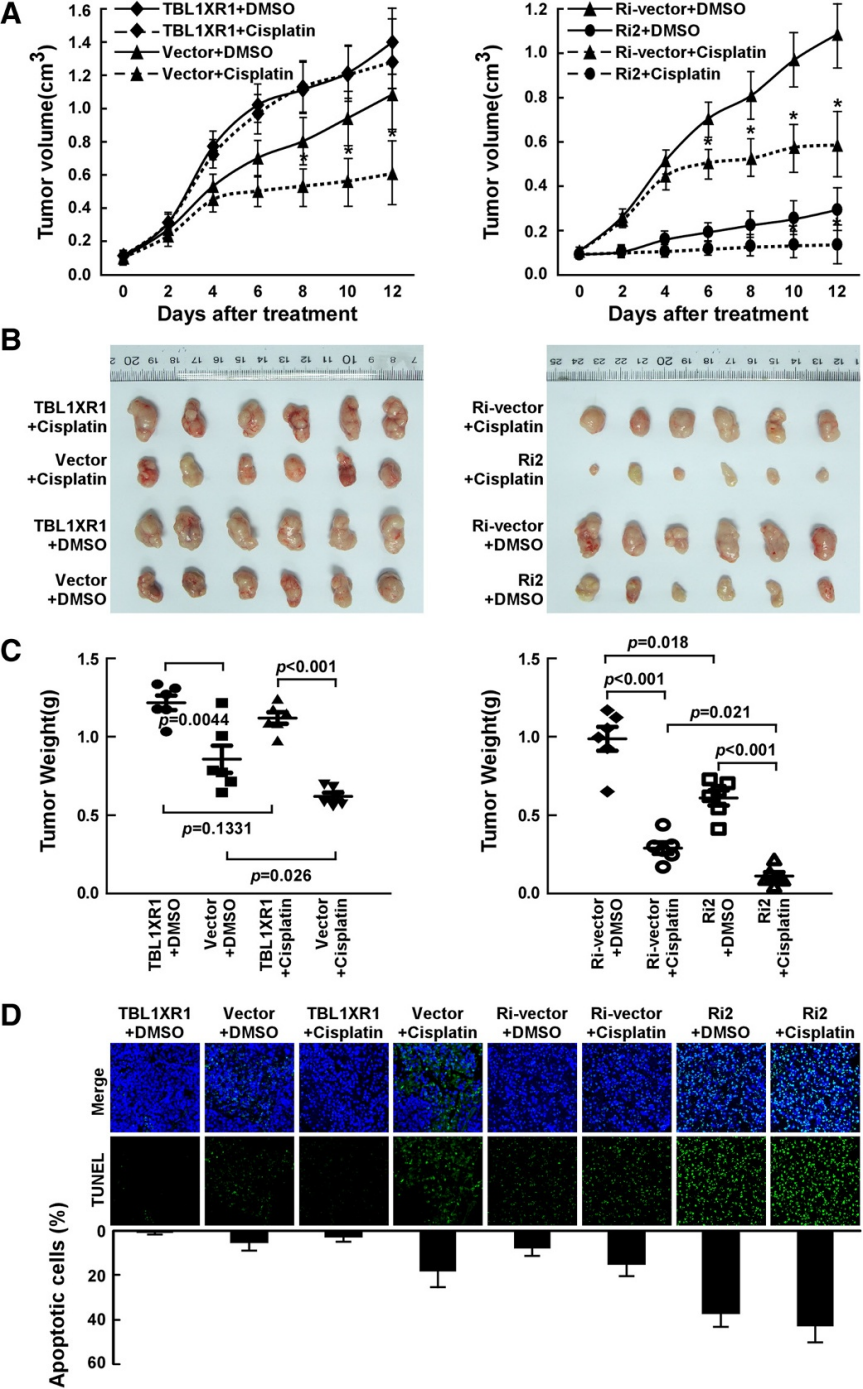
**The impact of TBL1XR1 expression on tumor growth** ***in vivo*** **.** The tumors formed by TBL1XR1-transduced CNE2 cells were larger than the vector control tumors. Conversely, the tumors formed by TBL1XR1-silenced cells were smaller than the tumors formed by the RNAi-vector cells. **(A)** Tumor volumes measured on the indicated days. Data points are presented as the mean tumor volume ± SD. **(B)** Representative images of the tumors from all mice in each group. **(C)** Weights of the tumors from all mice in each group. **(D)** Representative immunofluorescent images (Upper panel) and quantification (Lower panel) of TUNEL-stained cells in indicated tumors. The numbers of TUNEL-positive cells were counted from 10 random fields and presented as percentages of total cell numbers.

To examined the effect of TBL1XR1 on apoptosis *in vivo,* nude mice were subcutaneously injected with CNE2 cells. When tumors reached a volume of about 100 mm^3^, animals were randomly assigned to two groups and given an intraperitoneal injection of 100 ml DMSO (control) or cisplatin. Interestingly, the volumes and weights of tumors formed by the CNE2-TBL1XR1 cells were not significantly affected by cisplatin treatment (Figure 5A-C). However, tumors formed by vector control cells, or by cells with depleted endogenous TBL1XR1, exhibited a striking inhibition of tumor growth in terms of both tumor volume and weight after cisplatin treatment (Figure 5A-C). These results are strongly indicative of TBL1XR1-associated resistance to cisplatin, and are consistent with the other results.

To determine whether high TBL1XR1-expression alters cell survival within tumors, we analyzed tumors harvested from animals in indicated groups for apoptotic frequency. As shown in Figure 5D, consistent with above results, after cisplatin treatment, the percentage of apoptotic cells in tumors obtained from the CNE2-TBL1XR1 group was significantly reduced in comparison with that in tumors obtained from the other group, strongly suggesting a suppressive effect of elevated TBL1XR1 on cisplatin sensitivity within the NPC cells.

### TBL1XR1 activates NF-κB signaling pathway

**Figure 6:**
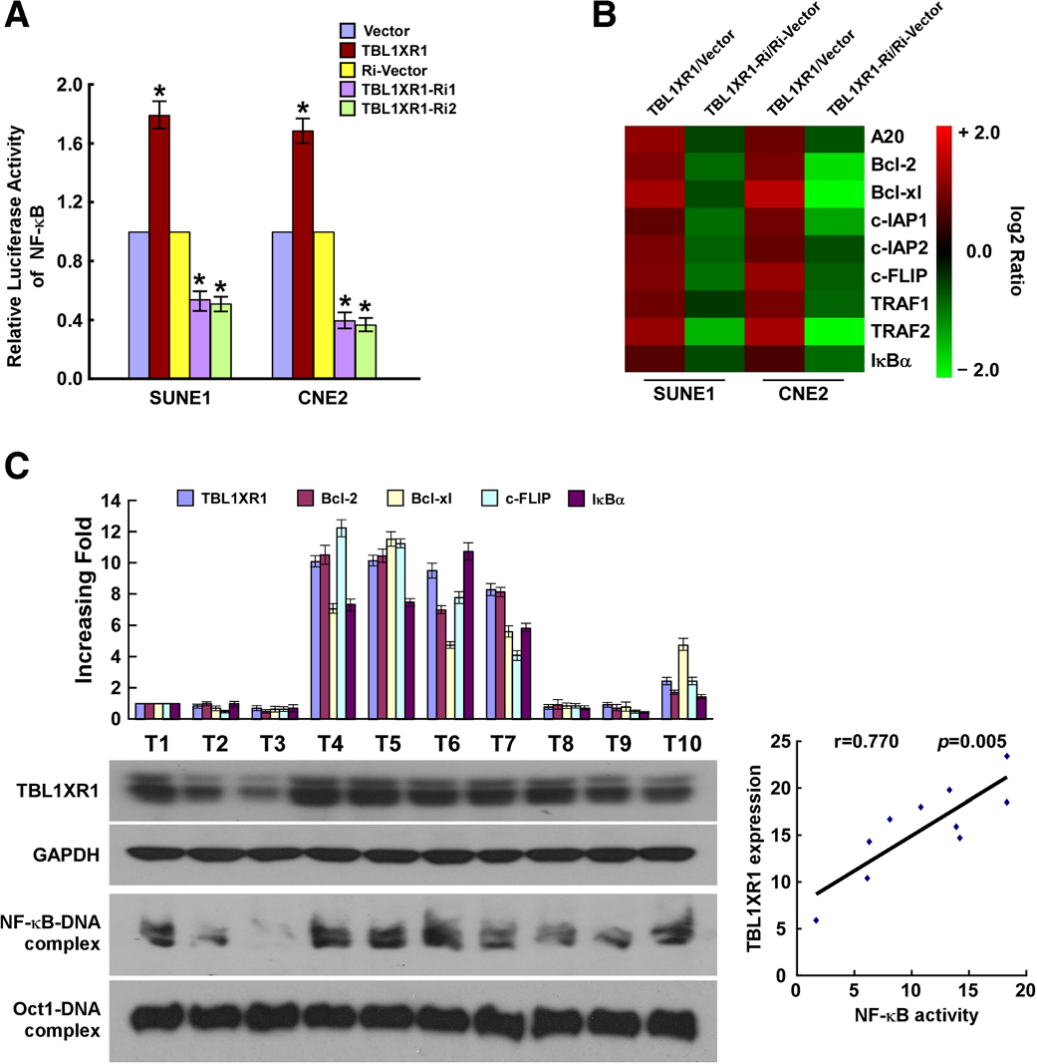
**TBL1XR1 activates NF-κB signaling pathway. (A)** Luciferase-reporter NF-κB activity in indicated cells. **(B)** Real-time PCR analysis indicating an apparent overlap between NF-κB-dependent gene expression and TBL1XR1-regulated gene expression. The pseudo color represents the intensity scale of TBL1XR1 vs Vector, or TBL1XR1 short hairpin RNA vs RNAi-vector, generated by a log2 transformation. **(C)** Analysis of expression and correlation of TBL1XR1 with Bcl-2, Bcl-xl, c-FLIP and IκB mRNA expression, as well as NF-κB activity in 10 freshly collected NPC biopsies.

TBL1XR1 is involved in multiple pathways, including the Wnt/β-catenin, Notch, NF-κB, and nuclear receptor pathways [10–13], The activation of NF-κB signaling is associated with anti-apoptotic properties, and we investigated whether TBL1XR1 promoted anti-apoptotic effects in NPC cells via this pathway. The NF-κB luciferase assay revealed that TBL1XR1 overexpression was accompanied by the downregulation of NF-κB and genes downstream of NF-κB (Figure 6A and B).

To establish the clinical relevance of this observation, TBL1XR1 expression and NF-κB activation was measured in 10 freshly collected clinical NPC samples. Real-time RT-PCR, Western blot and EMSA assays showed that TBL1XR1 protein levels were positively correlated with mRNA levels of several NF-κB downstream target genes, and also with NF-κB DNA binding. TBL1XR1 is upregulated in NPCs, activates the NF-κB signaling pathway and confers anti-apoptotic properties on these cells.

Moreover, when we further examined the effect of the Epstein–Barr virus latent membrane protein 1 (LMP1) on the expression levels of TBL1XR1 in SUNE1, CNE2 and C666 NPC cells. Our result showed that neither overexpressing nor silencing LMP1 had any influence on the mRNA and protein levels of TBL1XR1 (Additional file 1: Figure S1), implicating that the biological role of TBL1XR1 in NPC cells herein was EBV-independent.

## Discussion

The key finding in this report lies in, for the first time, the biological role of TBL1XR1 in NPC progression and chemotherapy resistance. TBL1XR1 mRNA and protein levels were both elevated in NPC cells *in vitro*, and this was also observed in clinical samples. In addition, in vitro assays indicated that TBL1XR1 promoted anti-apoptosis in NPC cells by activating NF-κB pathway, further cementing the role of TBL1XR1 as an oncogenic protein [14, 15].

Local recurrence and therapy resistance are the two main problems associated with the treatment of NPC patients. In this study, NPC cells were treated with cisplatin, a standard chemotherapeutic agent that can also enhance the effectiveness of radiotherapy when used in combination with others. The MTT, TUNEL and Annexin-V binding assays all showed that elevated TBL1XR1 expression markedly reduced the ability of cisplatin to kill NPC cells. Conversely, cells in which TBL1XR1 was downregulated were more sensitive to cisplatin.

The above results led us to believe that upregulation of TBL1XR1 may reduce cisplatin-induced apoptosis in NPC cells. Aberrant apoptotic signals is significantly involved in oncogenesis and tumor regression in numerous cancers, including NPC. Previous researchers have identified several factors associated with apoptosis. B-cell lymphoma 2 (Bcl-2), a classical anti-apoptosis protein, has been shown to be overexpressed in biopsy specimens from NPC patients, and Bcl-2 is predominately co-expressed with p53 in NPC [17–20]. The apoptotic inhibitor, survivin, has been found to be upregulated in NPC, and may serve as a potential prognostic marker for NPC patients [21–23]. Other molecules have also been reported to be correlated with the protection of NPC cells from apoptosis [24–29]. However, the exact mechanisms underlying the regulation of apoptosis in NPC need to be explored conclusively.

NF-κB signaling plays an vital role in cell survival by upregulating gene products that block apoptosis. TBL1XR1, a corepressor/coactivator exchange factor, has been reported to regulate switch between gene repression and gene activation in transcriptional regulation, and be involved in activation of mutiple signalling pathways, including NF-κB pathways [10]. Hoberg et al. proposed that TBL1XR1 function as an E3 ubiquitin ligase that recruits UbcH5 ubiquitin conjugating enzymes, and Ubc5-dependent targeting to the proteasome and removes SMRT from NF-κB binding region, subsequently replaces of SMRT with coactivators in a ligand-dependent manner [6]. Above studies suggested us that upregulation of TBL1XR1 in NPC, may promote resistance to cisplatin-induced apoptosis via stimulating the NF-κB pathway. The results of the NF-κB luciferase reporter assay demonstrated that TBL1XR1 was indeed involved. In addition, real-time RT-PCR results indicated that there was a strong positive correlation between the expression of NF-κB target genes and TBL1XR1 expression level in two independent NPC cell lines. This relationship held true in ten freshly collected clinical NPC samples, which emphasized the clinical relevance of our findings.

TBL1XR1 is located at 3q26.32. Though the chromosome region 3q21–q26.2 was previously reported to be frequently amplified in NPC tissues [30, 31]. By using the genomic PCR assay, we did not find a significant change of TBL1XR1 copy number in NPC tissues (Additional file 2: Figure S2), indicating that TBL1XR1 overexpression is not due to the genomic amplification. Interestingly, our results indicated that TBL1XR1 was expressed at a very low level in normal samples, but markedly in NPC samples. Moverover, TBL1XR1 mRNA and protein expression are significantly correlated, suggeting that TBL1XR1 might be upregulated at the transcriptional level. By analysis of the promoter region of TBL1XR1 using the CONSITE program, we found two typical responsive E-BOX elements of transcriptional factor c-myc, which has been reported to be overexpressed in NPC [32]. Meanwhile, CpG islands were also observed in TBL1XR1 promoter. Thus, it would be of great interest to further investigate whether upregulation of TBL1XR1 in NPC is attributed to c-myc transactivation or demethylation of CpG islands in NPC samples.

## Conclusion

In summary, this study has established clear links between the transcription cofactor TBL1XR1 and both NPC progression and chemotherapy resistance. Nevertheless, understanding the precise role of TBL1XR1 in the progression of NPC and activation of the NF-κB signaling will not only advance our knowledge of the mechanisms underlying NPC progression, but also help establish TBL1XR1 as a biomarker for clinical outcome and a potential therapeutic target in NPC.

## Materials and methods

### Cell lines

The normal nasopharyngeal epithelial cell line was cultured at 37°C and 5%CO_2_ with keratinocyte serum-free medium (Invitrogen, Carlsbad, CA) supplemented with epithelial growth factor, bovine pituitary extract, 120 μg/ml streptomycin and 120 μg/ml penicillin [33]. The human NPC cell lines, including C666, CNE1, CNE2, SUNE1, Hone1, HNE1, HK1, 5-8 F and 6-10B were grown in RPMI 1640 (Invitrogen) supplemented with 10% fetal bovine serum (HyClone, Logan, UT) and 100 μg/μL streptomycin, and 100 μg/μL penicillin in a humidified incubator containing 5% CO_2_ at 37°C.

### Patient information and tissue specimens

This study was conducted on a total of 105 paraffin-embedded NPC samples, which were histopathologically and clinically-diagnosed NPC patients treated at the Cancer Center, Sun Yat-sen University between April 2000 and January 2003. All 105 cases of NPC were undifferentiated non-keratinizing carcinoma with World Health Organization (WHO) type III pathology. The normal nasopharyngeal epithelial tissues were obtained from three patients undergoing nasopharyngeal mucosal biopsies, who were subsequently diagnosed with chronic inflammation of nasopharyngeal mucosa. For the use of these clinical materials for research purposes, prior patient consent and approval from the Ethics Committees of the Cancer Center, Sun Yat-sen University in advance of the study.

### Plasmids and transfections

pMSCV/TBL1XR1-overexpressing human TBL1XR1 was generated by subcloning the PCR-amplified human TBL1XR1 coding sequence into pMSCV vector. To silence endogenous TBL1XR1, two TBL1XR1 small hairpin RNA (shRNA)s were designed and cloned into the pSuper-retro-puro vector to generate pSuper-retro-TBL1XR1-RNAis, respectively [34]. Retroviral production and infection were performed as described previously [34]. Stable cell lines-expressing TBL1XR1 or TBL1XR1 RNAis were passaged and harvested after selection for 10 days with 0.5 μg/ml puromycin medium.

### RNA extraction, reverse transcription, and real-time PCR

Total RNA from cultured cells and fresh tissues were extracted using the Trizol reagent (Invitrogen) according to the manufacturer’s instruction. The extracted RNA was pretreated with RNase-free DNase, and about 2ug RNA from each sample was used for cDNA synthesis with iScriptcDNA Synthesis Kit (Bio-Rad Laboratories, Hercules, CA). Real-time PCR primes were designed using the Primer Express Software Version 2.0 and the primer sequences are: TBL1XR1 forward primer: GAATTTCCTTGTGCCTCCAT; TBL1XR1 reverse primer: TGCAACTGAATATCCGGTCA; Glyceraldehyde-3-phosphate dehydrogenase (GAPDH) forward prime: 5′-GACTCATGACCACAGTCC ATGC-3′; GAPDH reverse primer: 5′-AGAGGCA GGGATGATGTTCTG-3′. Expression data were normalized to the geometric mean of housekeeping gene GAPDH to control the variability in expression levels and calculated as 2^-[(Ct of TBL1XR1) – (Ct of *GAPDH*)]^ , where C_t_ represents the threshold cycle for each transcript.

### Western blotting

Western blot analysis was performed according to standard methods as described previously [34], using anti-TBL1XR1 rabbit polyclonal antibody (1:3,000; Sigma). Blot membranes were stripped and reprobed with anti-GAPDH antibody (1:1,000; Sigma) as a loading control.

### Immunohistochemistry

Immunohistochemistry staining was carried out using Histostain-Plus Kits (Invitrogen) following the manufacturer’s protocols. Two independent pathologists blinded to the clinical parameters conducted the staining index (SI) for TBL1XR1 expression. The staining results were scored based on the following criteria: (i) percentage of positive tumor cells in the tumor tissue: 0 (0%), 1 (1%–25%), 2 (26%–50%), 3 (51%–75%) and 4 (76%–100%); (ii) staining intensity: 0 (no staining), 1 (weak staining = light yellow), 2 (moderate staining = yellow brown), and 3 (strong staining = brown). The SI was calculated as staining intensity score × proportion of positive tumor cells (range from 0 to 12) [34]. An optimal cutoff value was identified: the SI > 6 was used to define as TBL1XR1 high expression while SI ≤ 6 as TBL1XR1 low expression.

### MTT assay

5,000 cells were seeded in triplicate in 96-well plates, All cells were incubation with Cisplatin (Hospira Australia Pty Ltd., 5 μg/ml, 10 μg/ml, 15 μg/ml, 20 μg/ml) for 48 hours. 20 μl of 5 mg/ml MTT was added 4 hours prior to the time points when 150 ml of DMSO was added for each well. The absorbance was measured at 490 nm. All experiments were performed in triplicates.

### TUNEL assay

The DeadEndTM Fluorometric TUNEL System (Promega, Madison, WI) was used for TUNEL assay according to the manufacturer’s instruction. 3 × 10^4^cells were seeded on coverslips (Fisher Scientific) in 24-well plates. After 24 hours, all cells were incubation with Cisplatin, followed by washed once with cold PBS followed by fixation in freshly prepared 4% formaldehyde solution in PBS (pH 7.4) for 25 min at 4°C. The fixed slides were washed with PBS for 5 min and then permeabilized with 0.2% Triton X-100 solution in PBS for 5 min. After a 5-min rinse with PBS, and cells were covered with 100 μL Equilibration Buffer for 5 min, followed by a 60-min incubation with 2 x SSC at 37°C to terminate the reaction and a 5 min PBS wash. The samples were then stained in the dark with 1 μg/ml propidium iodide (PI) solution for 15 min. After a final wash with H_2_O for 5 min at ambient temperature and air-dry, samples were immediately analyzed under a fluorescence microscope using a standard fluorescein filter set to view the green fluorescence of fluorescein at 520 nm, the red fluorescence of PI at 620 nm.

### Annexin-V binding assay

The ApopNexinTM FITC Apoptosis Detection Kit (Millipore, Lake Placid, NY) was used to examine the apoptotic cells according to manufacturer’s instruction. 3 × 10^5^ cells were seeded in triplicate in 6-well plates. After 24 hours, all cells were incubation with Cisplatin, followed by washes with PBS and then with an Annexin-V binding solution. Subsequently, 150 μl of an Annexin-V antibody in Binding Buffer was added to each culture well and incubated for 15 min, followed by addition of 1.5 μL of PI at 1 mg/ml and a further incubation for 5 min. 10,000 cells were analyzed on a flow cytometer (FACSCalibur; BD Biosciences).

### Luciferase assay

3 × 10^4^ cells were seeded in triplicate in 24-well plates. After 24 h, 500 ng NF-κB luciferase reporter plasmids plus 5 ng of pRL-TK renilla plasmid (Promega) were co-transfected into NPC cells using the Lipofectamine 2000 reagent (Invitrogen) according to the manufacturer’s recommendation. Luciferase and renilla signals were measured 48 h after transfection using the Dual Luciferase Reporter Assay Kit (Promega) according to a protocol provided by the manufacturer. Three independent experiments were performed, and the data are presented as mean ± SD.

### Electrophoretic Mobility Shift Assay (EMSA)

Briefly, the nuclear proteins were harvested from fresh NPC biopsies using the NE-PER Nuclear protein extraction kit (Pierce Biotechnology) according to the manufacturer’s instructions. The NF-κB binding probe was synthesized with 5′ biotin labels. Binding reactions were equivalent in that 20 fmol probe was incubated with 5.0 μg nuclear proteins, and then subjected to non-denaturing polyacrylamide gel electrophoresis. The NF-κB-DNA-binding complex shift was then dected using the LightShift Chemiluminescent EMSA Kit (Pierce Biotechnology). Oct-1-DNA-binding complexes served as a loading control. The DNA probes used were as following: NF-κB: sense, 5′-AGTTGAGGGGACTTTCCCAGGC-3′, antisense, 5′-GCCTGGG AAAGTCCCCTCAAC-3′; OCT-1: sense, 5′-TGTCGAATGCAAATCACTAGAA-3′, antisense, 5′-TTCTAGTGATTTGCATTCGACA-3′.

### In vivo experiment

Female BALB/c nude mice (4–5 weeks of age, 18–20 g) were purchased from the Animal Center of Guangdong Province and were housed in barrier facilities on a 12-hour light/dark cycle. All experiments were approved by the animal care committee at the Sun Yat-sen University Cancer Center. The BALB/c nude mice were randomly divided into 2 groups (12/group). One group of mice were inoculated subcutaneously with CNE2-TBL1XR1/ CNE2-Vector cells (1 × 10^6^ suspended in 100 mL sterile PBS) per mouse, another group of mice inoculated with CNE2- Ri-Vector /CNE2- TBL1XR1-Ri2 cells (1 × 10^6^ suspended in 100 mL sterile PBS) per mouse. Tumor volume was calculated using the equation (LxW^2^)/2. Mice were checked every 2 days for xenograft development. When tumors became palpable (about 100 mm^3^), each group of mice mice were randomly divided into 2 subgroups (6/group), followed by intraperitoneal injection of 100 mL vehicle (dimethyl sulfoxide, DMSO), Cisplatin (5 mg/kg), respectively, every2 days.

### Statistical analysis

All statistical analyses were carried out using the SPSS v.13.0 statistical software packages (SPSS Inc, Chicago, IL, USA). The correlation between TBL1XR1 expression and the clinicopathological characteristics was analyzed by the Chi-Square test. Survival curves were plotted by the Kaplan-Meier method and compared with the log-rank test. The differences between experimental conditions were compared using Student’s t tests. P ≤ 0.05 was considered statistically significant.

## Electronic supplementary material

Additional file 1: Figure S1: TBL1XR1 expression is independent of LMP1. **(A)** Overexpression of LMP1 in SUNE1 and CNE2 cell lines. Western blotting analysis of TBL1XR in SUNE1-vector, SNUE-LMP1, CNE2-vector and CNE2-LMP1 cells. GAPDH was used as loading control. **(B)** LMP1 knockdown was achieved by introducing specific shRNA in C666 cell line. **(C)** Real-time PCR analysis of TBL1XR in SUNE1-vector, SNUE-LMP1, CNE2-vector, CNE2-LMP1, C666-vector and C666-LMP1-Ri cells. TBL1XR1 expression levels are presented as fold changes relative to vector-control cells and normalized to GAPDH. * P ≤ 0.05. (TIFF 355 KB)

Additional file 2: Figure S2: The copy number of the TBL1XR1 gene was measured by a TaqMan Copy Number Assay. RPPH1 gene on chromosome 14 as a reference locus. (TIFF 55 KB)

## Abbreviations

TBL1XR1: Transducin β-like 1 X-linked receptor 1
NPC: Nasopharyngeal carcinoma
IHC: Immunohistochemical staining
TBLR1: Transducin β-like-related protein 1
TBL1: Transducin β-like protein 1
N-CoR: Nuclear receptor corepressor
SMRT: Silencing mediator for retinoid and thyroid hormone receptors
PPAR: Peroxisome proliferator-activated receptor
CGH: Comparative genomic hybridization
shRNA: Small hairpin RNA.
