# Supplementary figures and images for: Transducin β-like 1 X-linked receptor 1 suppresses cisplatin sensitivity in Nasopharyngeal Carcinoma via activation of NF-κB pathway
Source: Mol Cancer. 2014 Aug 22;13:195. doi: 10.1186/1476-4598-13-195 (PMC4158072; doi:10.1186/1476-4598-13-195)

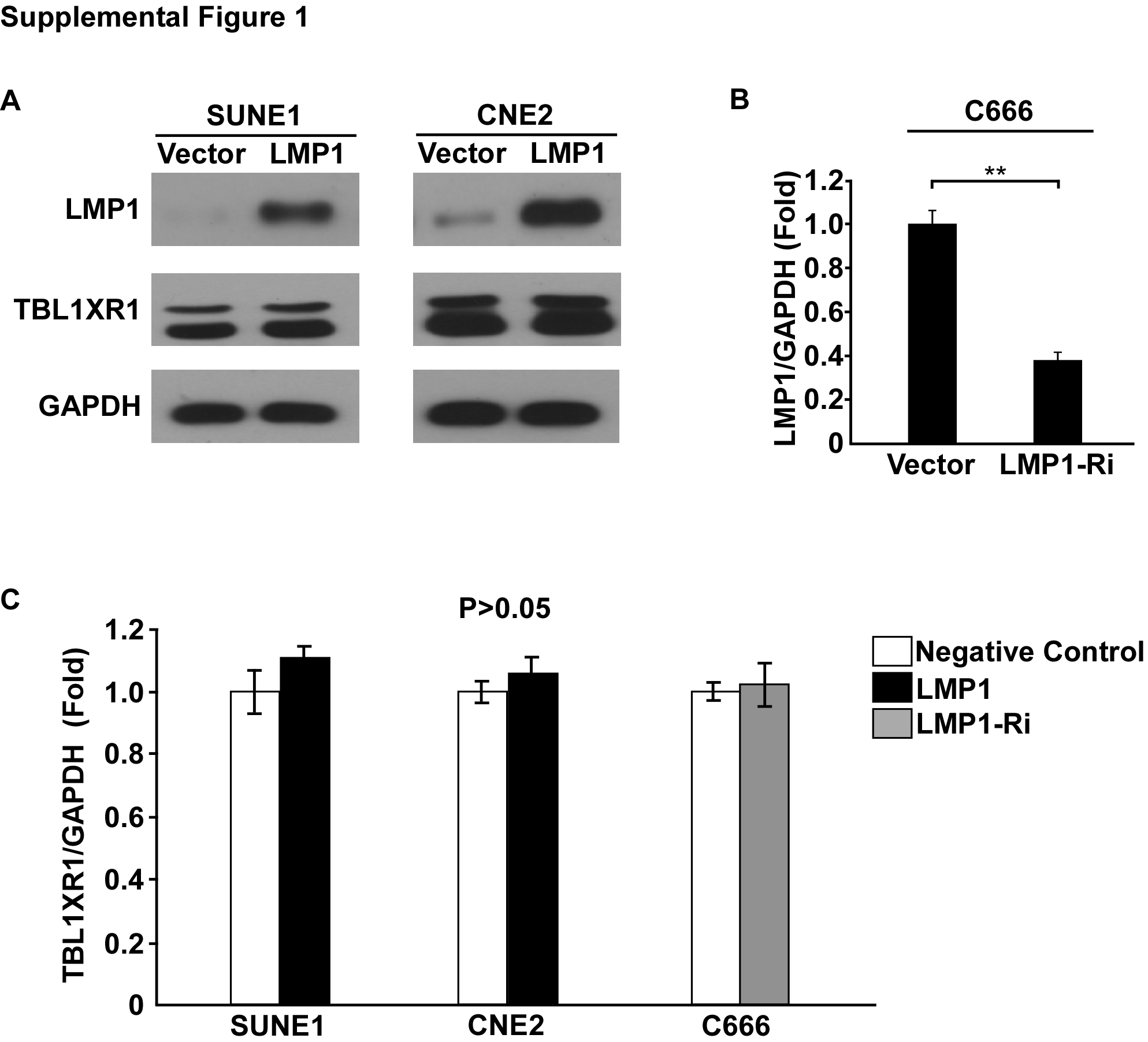

Supplement: Supplementary file 1 — Additional file 1: Figure S1: TBL1XR1 expression is independent of LMP1. (A) Overexpression of LMP1 in SUNE1 and CNE2 cell lines. Western blotting analysis of TBL1XR in SUNE1-vector, SNUE-LMP1, CNE2-vector and CNE2-LMP1 cells. GAPDH was used as loading control. (B) LMP1 knockdown was achieved by introducing specific shRNA in C666 cell line. (C) Real-time PCR analysis of TBL1XR in SUNE1-vector, SNUE-LMP1, CNE2-vector, CNE2-LMP1, C666-vector and C666-LMP1-Ri cells. TBL1XR1 expression levels are presented as fold changes relative to vector-control cells and normalized to GAPDH. * P ≤ 0.05. (TIFF 355 KB) [file 12943_2014_1398_MOESM1_ESM.tiff]

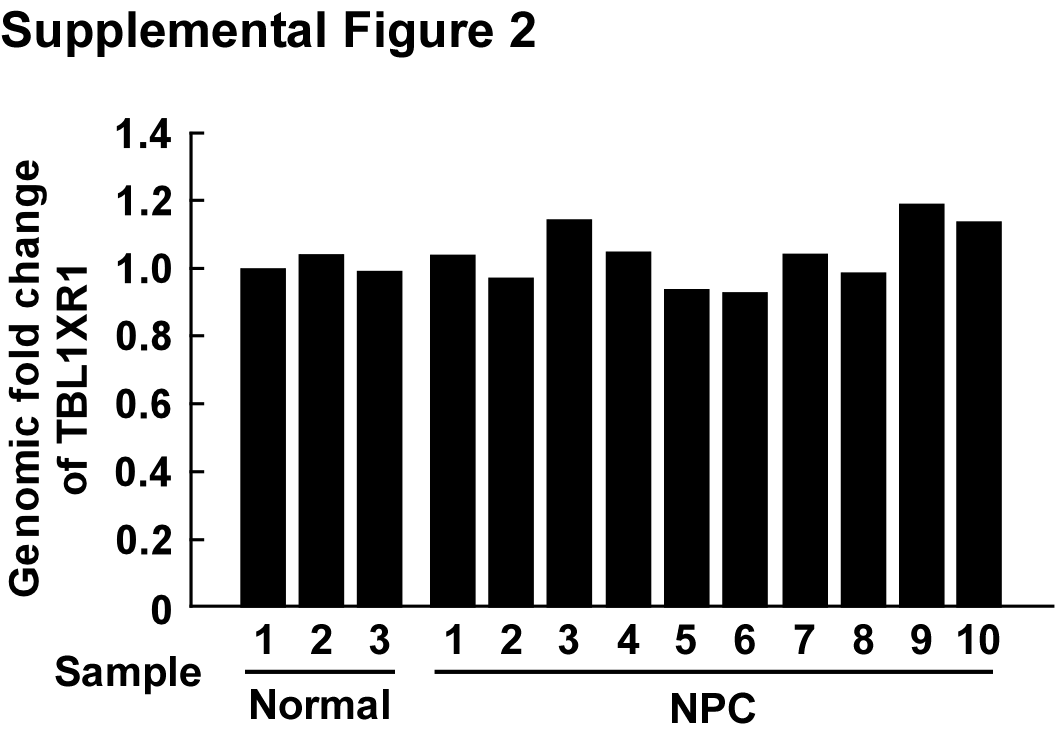

Supplement: Supplementary file 2 — Additional file 2: Figure S2: The copy number of the TBL1XR1 gene was measured by a TaqMan Copy Number Assay. RPPH1 gene on chromosome 14 as a reference locus. (TIFF 55 KB) [file 12943_2014_1398_MOESM2_ESM.tiff]
